# Unboosted atazanavir with lamivudine/emtricitabine for patients with long-lasting virological suppression

**DOI:** 10.7448/IAS.17.4.19811

**Published:** 2014-11-02

**Authors:** Alessia Carbone, Laura Galli, Alba Bigoloni, Simona Bossolasco, Monica Guffanti, Miriam Maillard, Elisabetta Carini, Stefania Salpietro, Vincenzo Spagnuolo, Nicola Gianotti, Adriano Lazzarin, Antonella Castagna

**Affiliations:** Infectious Diseases, San Raffaele Hospital, Milan, Italy

## Abstract

**Introduction:**

Unboosted atazanavir (ATV) including regimens have been investigated as a ritonavir-sparing simplification strategy. No data are available on removal of one NRTI in subjects effectively treated with unboosted atazanavir+2NRTIs. We present the 48-week virological efficacy and safety of unboosted atazanavir plus lamivudine (3TC) or emtricitabine (FTC) (lamivudine/emtricitabine/Reyataz^©^, LAREY Study).

**Materials and Methods:**

Single arm, prospective, pilot study on HIV-treated patients, HBsAg negative, with HIV-RNA<50 cps/mL since at least 2 years, who switched from ATV+2NRTIs to ATV 400 mg QD +3TC or FTC. Virological failure was defined as 2 consecutive values of HIV-RNA>50 cps/ml; viral blip was defined as a single HIV-RNA value>50 cps/ml not subsequently confirmed. Results as median (IQR). Changes between baseline (BL) and week 48 assessed by the Wilcoxon signed rank test.

**Results:**

Forty patients enrolled: 75% males, 51 (47–54) years, 14% HCV co-infected, infected with HIV since 16 (9–21) years, on antiretroviral therapy since 13 (5–16) years, with a nadir CD4+ of 254 (157–307) cells/mm^3^, virologically suppressed since 4.2 (2.2–5.4) years; 53 patients switched from a tenofovir (TDF)-based regimens; ATV was associated with 3TC in 83% patients. No virological failures or discontinuations were observed; three patients had a single viral blip in the range 50–250 copies/mL; CD4+ increased from 610 (518–829) cells/mm^3^ at BL to 697 (579–858) cells/mm^3^ at week 48 [48-week change: 39 (−63/+160) cells/mm^3^ p=0.081]. Three clinical events were observed (one herpes zoster, one pneumonia, one syphilis) in absence of renal lithiasis, AIDS-defining or drug-related events or death. Overall, significant 48-week amelioration of ALP [BL: 83 (71–107) mg/dL; 48-week change: −15 (−27/−8) mg/dL p<0.0001] and CKD-EPI [BL: 100 (86–108) ml/min/1.73 m^2^; 48-week change: 1.5 (−3/+8) ml/min/1.73 m^2^, p=0.042] were observed. Patients switching from TDF (Table 1) significantly improved CD4+, lymphocytes, hepatic profile, renal profile and ALP; these patients had also a modest but significant decrease in haemoglobin.

**Conclusions:**

Switch from an unboosted atazanavir-based regimen to ATV+3TC or FTC regimen was effective and safe in this small sample, supporting the hypothesis of a potential two-steps de-intensification (removal of ritonavir and removal of one NRTI) in patients on long-lasting virological suppression.

**Table 1:** Laboratory characteristics according to switch from tenofovir or other NRTIs in the LAREY study

	Baseline	Switch from tenofovir 48-week change	p value^a^	Baseline	Switch from other NRTIs 48-week change	p value^a^	p value on 48-week change^a^ (TDF vs other NRTIs)
CD4+ (cells/mm^3^)	572 (528–814)	155 (+19/+197)	0.0008	642 (508–885)	−48 (−122/+4)	NS	0.002
Haemoglobin (mg/dL)	15.2 (14.1–16.3)	−0.2 (−0.9/+0.2)	0.048	14.6 (14.2–15.1)	0.1 (−0.1/+0.6)	NS	0.044
Lymphocytes (10^9^ cells/L)	2.1 (1.8–2.4)	0.3 (−0.1/+0.5)	0.015	2.5 (2.0–3.0)	−0.2 (−0.7/+0.1)	0.024	0.002
Neutrophil (10^9^ cells/L)	3.8 (2.9–5.7)	−0.2 (−1.0/0)	NS	4.0 (3.2–4.6)	−0.1 (−0.9/+0.5)	NS	NS
Cholesterol (mg/dL)	192 (181–208)	10 (−17/+39)	NS	201 (172–221)	−1 (−16/+14)	NS	NS
HDL-cholesterol (mg/dL)	39 (37–66)	1.5 (−6.5/+3)	NS	43 (38–52)	−1.5 (−6/+1)	NS	NS
LDL-cholesterol (mg/dL)	123 (101–141)	3 (−12/+12)	NS	123 (94–135)	−7.5 (−34/+28)	NS	NS
Triglycerides (mg/dL)	96 (84–202)	−10 (−23/+59)	NS	112 (80–280)	−17 (−62/−1)	NS	NS
Total bilirubin (mg/dL)	1.92 (1.18–2.71)	0.08 (−0.45/+1.23)	NS	1.62 (1.24–2.01)	0.19 (−0.16/+1.55)	NS	NS
Direct bilirubin (mg/dL)	0.33 (0.23–0.60)	0.07 (−0.31/+0.16)	NS	0.4 (0.36–0.49)	0.09 (−0.09/+0.31)	NS	NS
AST (U/L)	24 (15–32)	−1 (−9/+2)	NS	21 (18–27)	3 (+1/+5)	0.043	0.009
ALT (U/L)	40 (22–44)	−7 (−17/0)	0.003	29 (22–35)	4 (−1/+15)	0.040	0.001
APRI	0.25 (0.17–0.40)	−0.02 (−0.1/+0.3)	NS	0.25 (0.18–0.31)	0.05 (−0.02/+0.09)	NS	0.014
ALP (U/ml)	86 (81–108)	−22 (−29/−10)	<0.0001	75 (69–104)	−10 (−15/−2)	NS	0.004
CKD-EPI (ml/min/1.73 m^2^)	100 (87–107)	5 (−1/+9)	0.020	99 (85–110)	0 (−4/+6)	NS	NS
Creatinine (mg/dL)	0.85 (0.67–1.0)	−0.04 (−0.13/+0.04)	0.037	0.76 (0.73–0.89)	0 (−0.07/+0.05)	NS	NS

a By Wilcoxon signed rank test.

NS, not significant (p>0.05).

